# Identification and functional characterization of major gene *pcmfs*, controlling cap color formation in *Pleurotus cornucopiae*

**DOI:** 10.1128/aem.01894-24

**Published:** 2025-02-27

**Authors:** Yan Zhang, Siyu Xu, Yuting Li, QianQian Zhang, Wei Wang, Zhuang Li

**Affiliations:** 1 Shandong Provincial Key Laboratory of Agricultural Microbiology, College of Plant Protection, Shandong Agricultural University 34734 , Tai'an, China; Royal Botanic Gardens, Surrey, United Kingdom

**Keywords:** oyster mushroom, cap color, major gene, major facilitator superfamily

## Abstract

Oyster mushrooms are grown commercially worldwide, especially in many developing countries, for their easy cultivation and high biological efficiency. Cap color is an important commercial trait for oyster mushrooms. Little is known about the genetic mechanism of the cap color trait in oyster mushrooms, which limits molecular breeding for the improvement of cap color-type cultivars. In this study, an important candidate gene, *pcmfs*, for cap color in the oyster mushroom *Pleurotus cornucopiae* was identified based on the results of QTL (quantitative trait loci) mapping and comparative transcriptome analysis of our previous research. The *pcmfs* gene belonged to major facilitator superfamily (MFS) and was localized to the cell membrane. Expression pattern analysis and overexpression experiment demonstrated that *pcmfs* played an important positive role in cap color formation, with high expression levels leading to dark cap color. To our knowledge, this is the first reported gene that may be involved in the melanin transport in edible fungi. The results will enhance our understanding of the genetic basis for cap color formation in oyster mushrooms, ultimately facilitating the targeted molecular breeding of this phenotypic trait.

**IMPORTANCE:**

Oyster mushrooms are widely cultivated worldwide, particularly in developing countries, owing to their straightforward cultivation requirements and high biological efficiency. Cap color represents a significant commercial trait of oyster mushrooms. Despite its significance, the genetic basis of this trait remains poorly understood, limiting progress in molecular breeding to diversify cap color variants. Bridging this knowledge gap could improve the market appeal and consumer satisfaction of these cultivars by facilitating targeted breeding strategies. In our previous research, a major QTL of cap color in oyster mushroom *P. cornucopiae* was mapped and DEGs (differentially expressed genes) between the dark strains and white strains were identified. Based on this, the candidate gene for cap color *pcmfs* was further mined. The *pcmfs* gene, belonging to the major facilitator superfamily (MFS), is localized to the cell membrane. Expression pattern analysis and overexpression experiments have shown that *pcmfs* plays a significant role in cap color formation. To our knowledge, this is the first reported gene that may be involved in the melanin transport in edible fungi. The results contribute to elucidate the genetic mechanisms governing cap color formation in mushrooms, advancing targeted molecular breeding for this trait.

## INTRODUCTION

*Pleurotus* mushrooms, also known as oyster mushrooms, are cultivated commercially worldwide due to their simple cultivation, high biological efficiency, and significant nutritional and medicinal properties, leading to increased interest in recent years (1, 2). Economically valuable species include *Pleurotus ostreatus*, *Pleurotus cornucopiae*, *Pleurotus citrinopileatus*, *Pleurotus djamor*, *Pleurotus pulmonarius*, and *Pleurotus cystidiosus* (3, 4). Notably, *P. cornucopiae* is a major species produced and consumed in China (5–7). Cap color is a critical commercial trait of oyster mushrooms, ranging from white, black, pink, to yellow. Dark-cap oyster mushrooms are more and more popular, based on consumers’ preferences, but these dark varieties are rare on the market (8). Breeding superior cultivars of oyster mushrooms with darker caps is, therefore, needed. However, little is known about the genetic mechanism of the cap color trait in oyster mushrooms, which limits molecular breeding for the improvement of cap color-type cultivars.

Previous research has established that the pigment in oyster mushrooms is melanin. Furthermore, it has been confirmed that the pigments in various edible fungi, including *Auricularia auricula* (9), *Armillaria cepistipes* (10), and *Agaricus bisporus* (11), are also melanin. Thus, melanin is recognized as an important pigment type in fungi (12, 13). Melanin is typically synthesized intracellularly, followed by its transport to the extracellular space, where it matures into melanin granules that accumulate on the cell wall (14–16). Recent advancements in the study of functional genes associated with fungal melanin biosynthesis have yielded substantial progress (17). The tyrosinase (*tyr*) pathway plays a significant role in melanin synthesis. The *tyr* gene encodes the tyrosinase protein (E.C. 1.14.18.1), which functions as the rate-limiting enzyme in melanin synthesis (18, 19). Tyrosinase is a metalloprotein with an active site containing two copper ions and exhibits dual enzymatic activities, acting as both a hydroxylase and an oxidase. In the synthesis pathway of melanin, this enzyme is critical for two key rate-limiting steps: catalyzing the hydroxylation of L-tyrosine to L-DOPA and facilitating the oxidation of L-DOPA to dopaquinone (20). Fan et al. found that tyrosinase (*MoTyr*) was required for melanin synthesis and appressorium development in *Magnaporthe oryzae*. The loss of *MoTyr* resulted in a significant decrease in melanin synthesis (21). Weijn et al. discovered that tyrosinase catalyzes two distinct enzymatic reactions to synthesize melanin in *Agaricus bisporus* through bioinformatics analysis (11). Furthermore, the PKS pathway is important for fungal melanin biosynthesis. The early melanin synthesis enzymes PksP, Ayg1, Arp1, and Arp2 are recruited into endosomal vesicles, where the synthesis of early melanin is completed. Subsequently, the late melanin synthesis enzymes Abr1 and Abr2 are incorporated into these endosomal vesicles, forming multivesicular bodies (22). These multivesicular bodies are then secreted outside the cell membrane, where melanin synthesis continues, leading to the formation of mature melanin granules that ultimately accumulate on the cell wall (23, 24). In comparison to filamentous fungi, the genetic background of oyster mushrooms is more complex, which has resulted in slower progress in the investigation of genes associated with its coloration. Current research efforts have primarily been concentrated on the identification of QTL regions related to cap color through QTL mapping analysis (25, 26). Im et al. detected 12 QTL for cap color in *Pleurotus eryngii* across 5 linkage groups (LG1, LG4, LG5, LG6, and LG10) (27). LM et al. found that QTL clusters on LG5 and LG10 highlight the significance of these genomic regions for cap color traits in *P. eryngii* (28). Zhang et al. discovered that QTL for cap color in *P. ostreatus* were located on LG4 and LG10, explaining 9.93% and 7.26% of the phenotypic variation, respectively (29).

In previous research, a dark-colored strain *of P*. *cornucopiae* CCMSSC00406 and a white-colored strain *of P*. *cornucopiae* CCMSSC00358 were used as the parental lines to construct phenotypic segregation populations. A major QTL for cap color (0.8 Mb) was identified on chromosome 7 using traditional QTL analysis (29), BSA-seq (bulked-segregant analysis sequencing) analysis, and XP-GWAS (extreme-phenotype genome-wide association studies) analysis (30, 31). In order to further identify candidate genes, comparative transcriptome analysis was applied to find DEGs (differentially expressed genes) between the dark strains and white strains. As a result, *pctyr* was selected as an important candidate gene *pctyr* encoded the tyrosinase, which serves as the rate-limiting enzyme in the melanin synthesis pathway (32, 33). Expression patterns analysis and genetic transformation experiment proved that high expression level of *pctyr* led to the dark cap color, which confirmed that *pctyr* was a key gene in cap color formation (34).

In this study, the gene controlling cap color of oyster mushroom was further mined based on the results of QTL mapping and comparative transcriptome analysis of previous research. As a result, *pcmfs* was identified as another key candidate gene. Functional analysis demonstrated that *pcmfs* plays a critical role in the cap color formation in *P. cornucopiae*. Unlike the previously identified *pctyr* gene, *pcmfs* might play a significant role in the extracellular transport of melanin granules to the cell wall. To our knowledge, this is the first reported gene that may be involved in the melanin transport in edible fungi. The result will enhance our understanding of the genetic basis for cap color formation in oyster mushrooms and will facilitate molecular breeding for cap color.

## RESULTS

### Identification of the candidate gene associated with cap color

In our previous research, a major QTL associated with cap color in *P. cornucopiae* was identified through a combined analysis of traditional QTL mapping, BSA-seq, and XP-GWA. The major QTL was located within a 0.80 Mb region on chromosome 7 (chr7: 0.57–1.37 Mb). In this region, 187 genes were annotated in the reference genome of *P. cornucopiae* strain CCMMSSC006 (NCBI accession WQMT00000000). Meanwhile, comparative transcriptome analysis was performed using 5 dark-colored strains and 5 white strains, and 134 DEGs genes were identified (34).

**Fig 1:**
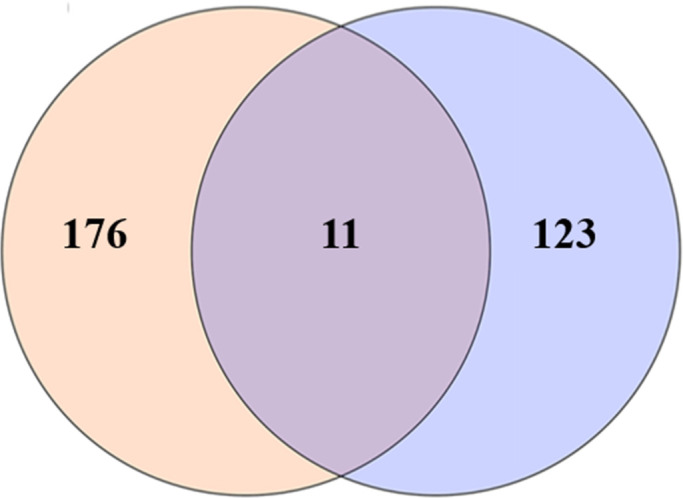
Venn diagram of genes in major QTL region for cap color and genes differentially expressed between the dark strains and white strains. The left circle represents 187 (176 + 11) genes located in the major QTL region for cap color; the right circle represents the 134 (123 + 11) differentially expressed genes (DEGs) selected between the dark strains and white strains by comparative transcriptome analysis. The overlapping number stands for 11 common gene between the above two groups.

A Venn diagram analysis was conducted on the 187 genes within the major QTL region and the 134 DEGs. The analysis revealed that only 11 differentially expressed genes were located within the major QTL region, comprising 7 upregulated and 4 downregulated genes (Fig. 1). Significant differences in the expression levels of the genes *g4221* and *g4215* have been observed between the dark-colored and white strains. The gene *g4221*, named *pctyr*, has been confirmed to play a crucial role in the cap color formation of *P. cornucopiae* in our previous research. Additionally, the FPKM value of *g4215* in the dark-colored strains gene was over 1,000 times higher than that in the white strains, suggesting that *g4215* may be another key gene controlling cap color formation (Table 1).

**TABLE 1:** Differentially expressed genes in the QTL region

Name	FPKM										
	CCMSSC 00406	F2-48	F2-126	F2-131	F2-133	CCMSSC 00358	F2-14	F2-51	F2-70	F2-71	Regulated
g4215	164.17	73.92	59.04	76.78	71.77	0.18	0.17	0.12	0.11	0.14	Up
g4218	90.87	47.46	46.55	46.25	42.04	14.74	14.65	12.61	13.09	18.28	Up
g4216	35.20	11.57	15.14	19.61	19.73	4.39	5.04	4.56	4.36	5.22	Up
g4214	8.56	3.72	3.89	4.21	4.90	1.13	1.87	2.11	1.93	2.87	Up
g4220	12.80	6.97	9.47	6.38	10.76	4.83	5.96	5.13	6.11	6.08	Up
g4221	201.07	76.00	66.52	92.00	59.47	0.38	0.33	0.26	0.55	59.47	Up
g8876	23.15	27.14	32.83	13.30	15.23	7.41	3.39	2.14	1.58	2.40	Up
g2436	1.84	2.05	2.55	2.44	2.39	3.83	25.69	14.15	14.13	9.71	Down
g6221	16.56	11.29	12.21	18.61	13.73	85.22	93.01	88.39	77.92	86.84	Down
g4279	20.86	31.01	34.47	24.44	35.36	60.37	64.76	69.46	46.20	98.69	Down
g4284	0.98	2.17	2.65	1.09	2.32	4.45	5.59	4.50	5.52	7.98	Down

**Fig 2:**
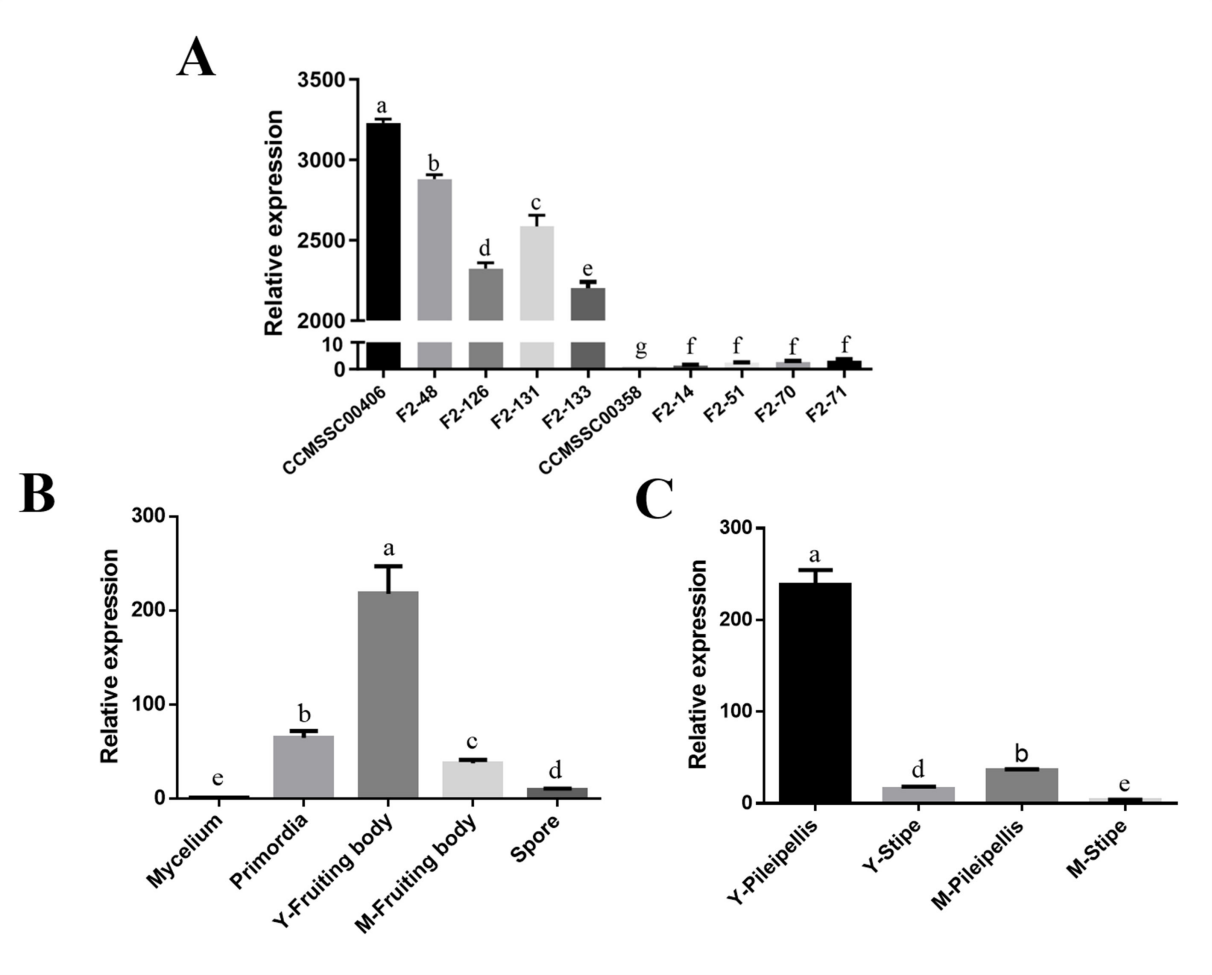
Expression pattern analysis of the *g4215* gene. (**A**) Expression levels of the *g4215* gene in dark strains (CCMSSC00406, F2-48, F2-126, F2-131, F2-133) and white strains (CCMSSC00358, F2-14, F2-51, F2-70, F2-71), with *g4215* expression in the CCMSSC00358 strain defined as 1. (**B**) Expression pattern of the *g4215* gene in different developmental stages of CCMSSC00406 (Y-Fruiting body = young fruiting body; M-Fruiting body = mature fruiting body), with *g4215* expression in the mycelium stage defined as 1. (**C**) Expression pattern of *g4215* in different parts of CCMSSC00406 fruiting bodies (Y-Pileipellis = young cap epidermis; Y-Stipe = young stipe; M-Pileipellis = mature cap epidermis; M-Stipe = mature stipe), with *g4215* expression in the mature stipe defined as 1. Data are presented as the mean ± SD (*n* = 3). Statistical significance is represented by different letters corresponding to *P* < 0.05 based on the Duncan’s test.

To verify the gene function, we investigated the expression patterns of *g4215* with qPCR in strains with dark and white caps, at various developmental stages, and in different parts of fruiting bodies. Expression levels of *g4215* in the dark strain CCMSSC00406 were low in mycelia but increased sharply in primordia and peaked in young fruiting bodies. The expression was significantly downregulated during the maturation of fruiting bodies and showed a low expression level in basidiospores (Fig. 2A). The expression levels of the *g4215* gene also exhibited significant differences between the pileipellis and stipe. Regardless of whether the fruiting body was in the Y-fruiting body or M-fruiting body stage, the expression levels of *g4215* in the pileipellis were significantly higher than those in the stipe (Fig. 2B). Overall, the expression of *g4215* gene throughout all developmental stages and different parts of the fruiting bodies showed a good correlation with cap color of *P. cornucopiae*. In addition, expression levels of the *g4215* in pileipellis from five dark-colored strains (parental strain CCMSSC00406 and F2-48, F2-126, F2-131, F2-133) and five white strains (parental strain CCMSSC00358 and F2-14, F2-51, F2-70, F2-71) were detected. The results revealed that *g4215* expression was extremely low or nearly undetectable in the white strains, whereas significantly higher expression levels were observed in the dark-colored strains, with differences reaching over 3,000-fold (Fig. 2C). This substantial difference provided further evidence that the *g4215* gene played a critical role in the cap color formation.

### Identification and analysis of the *pcmfs* gene

**Fig 3:**
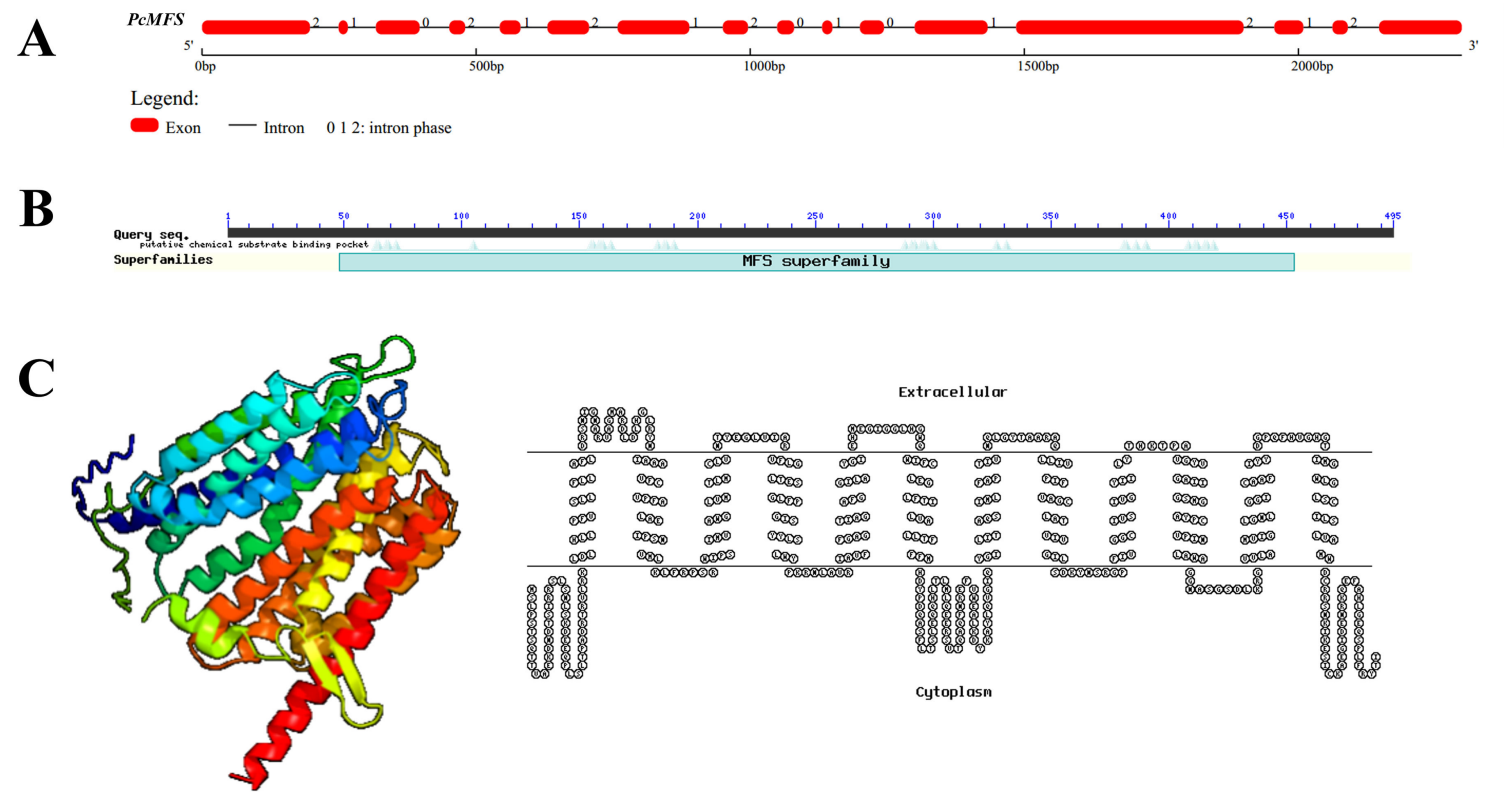
Sequence and protein structure analysis of the *g4215* gene. (**A**) Gene structure of *pcmfs*; (**B**) conserved domain analysis of *pcmfs*; (**C**, left) schematic representation of the 3D structure of the PCMFS protein; (**C**, right) prediction analysis of the transmembrane domains of *pcmfs*.

The physical position of *g4215* in the genome of CCMSSC00406 is at bp 848,183 to 850,480 on chromosome 7. The full length of the *g4215* gene is 2,298 bp, and it consists of 16 exons and 15 introns. *g4215* has a coding sequence of 1,488 bp and encodes a protein of 495 amino acid residues (Fig. 3A; GenBank accession number PQ757328). Conserved domain analysis revealed that *g4215* contains a major facilitator superfamily (MFS) conserved domain (Fig. 3B), and it was named *pcmfs*. To gain further insights into the function of the *pcmfs* gene, its protein structure was predicted using PHYRE2 and HMMTOP software. The results indicated that the PCMFS protein possesses 12 transmembrane α-helices, consistent with the characteristic protein structure of the MFS (Fig. 3C). This protein structure suggests that the *pcmfs* gene likely functions as a transmembrane transporter.

### Function analysis of the *pcmfs* gene

**Fig 4:**
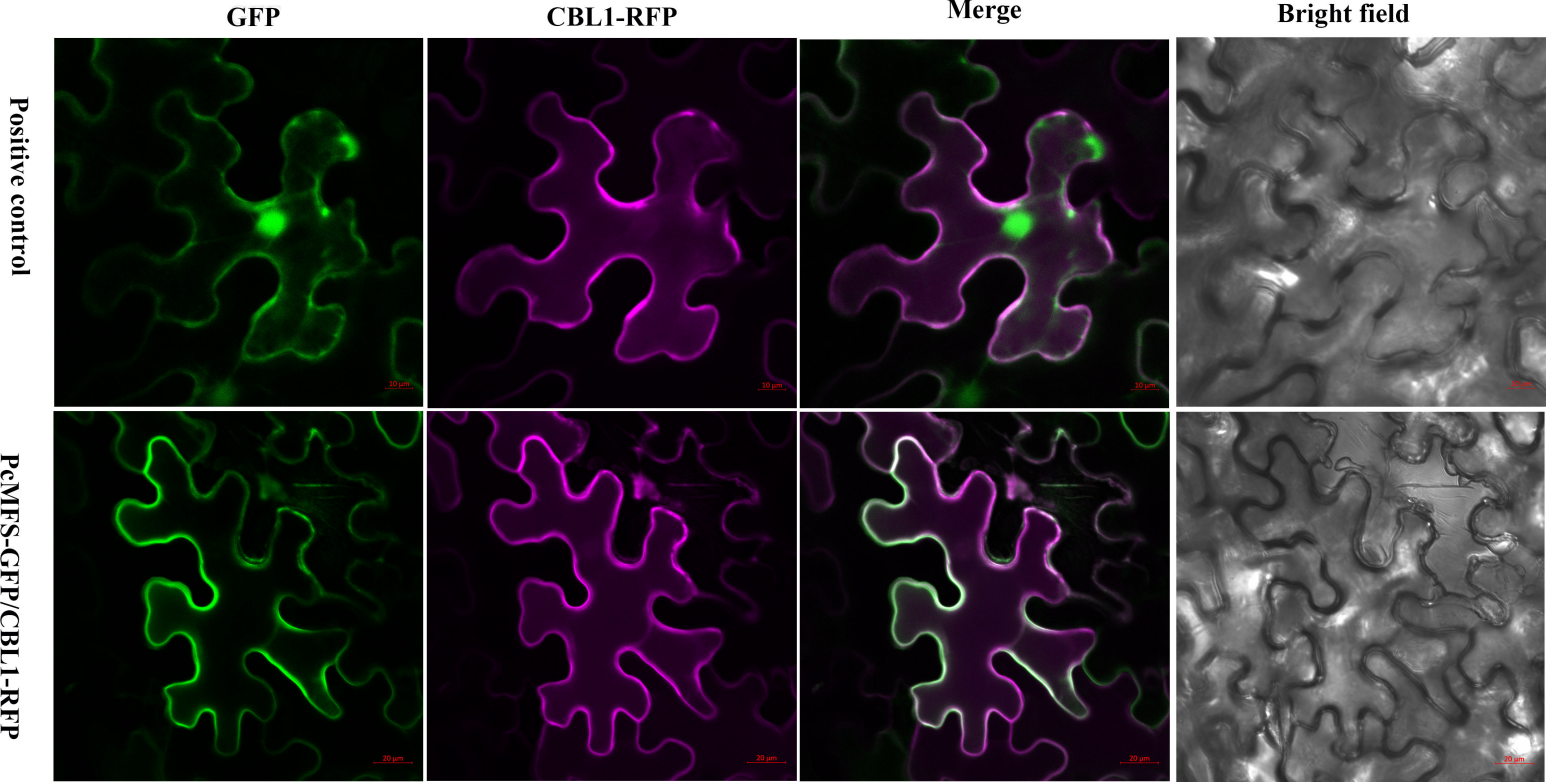
Subcellular localization of PCMFS protein. Both GFP localization analysis and CBL1-RFP localization analysis prove that PCMFS protein is localized to the cell membrane.

Bioinformatics analysis using Cell-PLoc (http://www.csbio.sjtu.edu.cn/bioinf/Cell-PLoc-2/) predicted that *pcmfs* located in cell membrane. To confirm the prediction, we cloned the *pcmfs* gene and fused to the N-terminal of green fluorescent protein (GFP) and CBL1-RFP (a membrane localization protein), respectively. The above vectors were transiently expressed in leaves of tobacco, and fluorescence analysis showed that PcMFS-GFP were distributed in the cell membrane and absent in the nucleus compared to the positive control. Meanwhile, PcMFS-CBL1-RFP were distributed in the cell membrane and signaled overlap with PcMFS-GFP signals (Fig. 4). Taken together, these results indicate that *pcmfs* is localized to the cell membrane.

**Fig 5:**
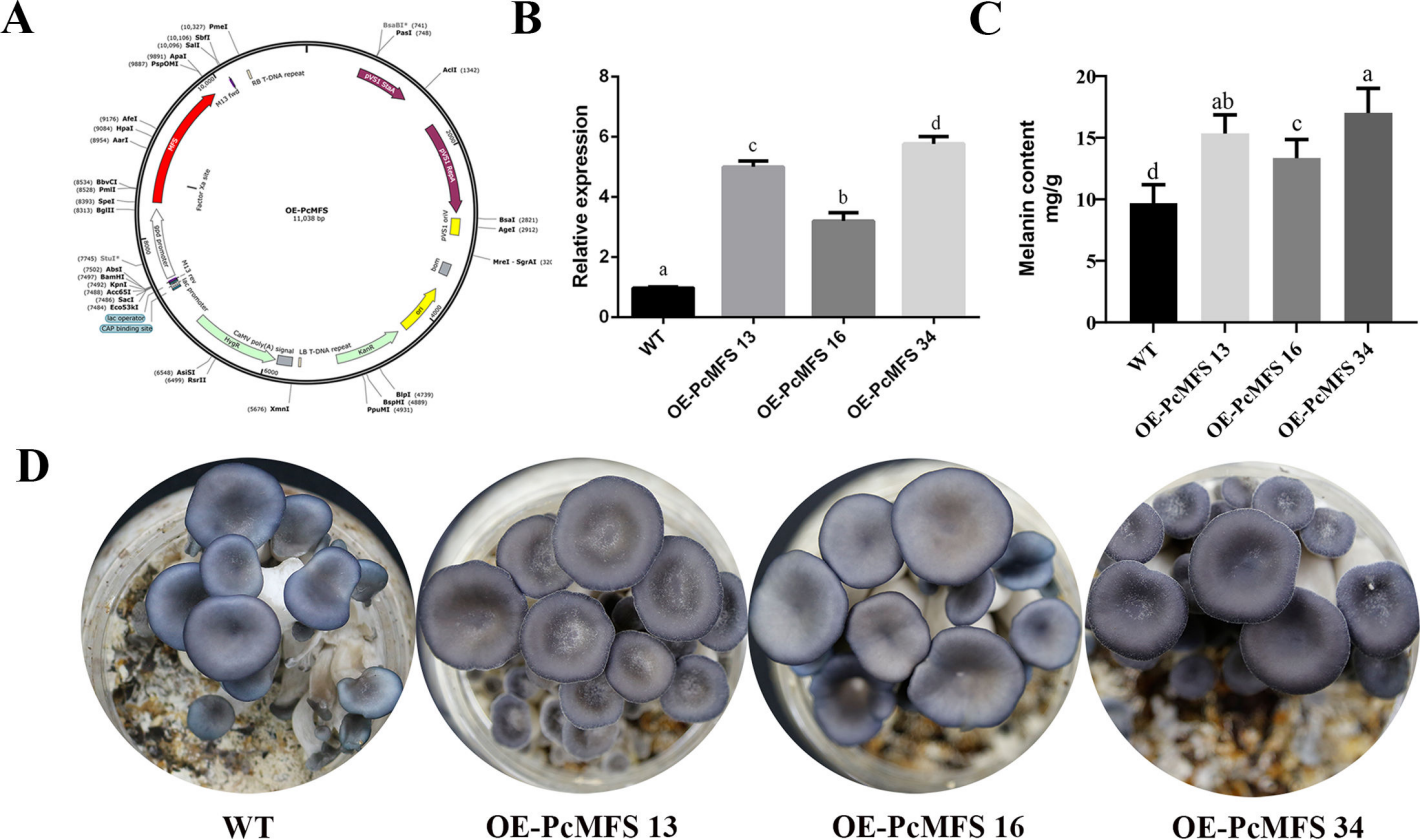
Construction of OE-PcMFS transformants and cap color phenotype images. (**A**) Plasmid map of OE-PcMFS; (**B**) expression levels of the *pcmfs* gene in OE-PcMFS transformants. The expression level of *pcmfs* in wild-type strain is defined as 1; (**C**) Melanin content of wild-type strain and OE-PcMFS transformants; (**D**) Cap color phenotype images of wild-type strain and OE-PcMFS transformants. Data are presented as the mean ± SD (*n* = 3). Statistical significance is represented by different letters corresponding to *P* < 0.05 based on the Duncan’s test.

To further analyze the role of *pcmfs* in cap color formation, the dark-colored strain CCMSSC00406 was used as the wild type (WT), and *pcmfs* overexpression (OE) transformants were generated using agrobacterium-mediated transformation. The plasmid map for the OE-PcMFS onstruct is shown in Fig. 5A. The expression levels of the *pcmfs* gene in the transformants were assessed using qPCR. The results indicated that the expression levels of *pcmfs* in the overexpression strains were upregulated significantly. The expression of *pcmfs* of strains OE*-*PcMFS 13, OE-PcMFS 16, and OE-PcMFS 34 was 4.62, 3.89, and 5.98 times as high as that of the WT strain, respectively (Fig. 5B). There were no significant differences observed between the *pcmfs* overexpression strains and WT strains in terms of mycelia growth, mycelia color traits, and fruiting body development rate. Fruiting experiment revealed that the cap color of the overexpression strains was darker than that of WT strains (Fig. 5C). Meanwhile, the overexpression of *pcmfs* resulted in an increase in melanin content. The melanin content of strains OE-PcMFS 13, OE-PcMFS 16, and OE-PcMFS 34 was 1.59 times, 1.38 times, and 3.96 times that of the WT strain, respectively. The results confirmed that the overexpression of *pcmfs* increased the content of melanin, leading to a darker cap color, which indicated that the *pcmfs* gene plays a pivotal role in cap color formation.

### Polymorphism analysis of the *pcmfs* gene sequence

**Fig 6:**
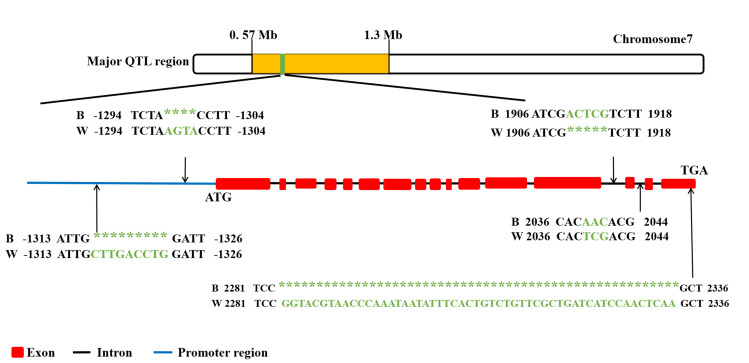
Polymorphism analysis of genic and promoter region of *pcmfs*.

To detect the sequence differences of *pcmfs* between white and dark strains, we compared the full-length sequences of the *pcmfs* gene and the 2,000 bp promoter sequences from five white strains (parental strain CCMSSC00358, F2-14, F2-51, F2-70, F2-71) with five dark strains (parental strain CCMSSC00406, F2-48, F2-126, F2-131, F2-133). The results showed that the most pronounced difference is observed in the final exon region, where the white strain exhibits a 53 bp sequence insertion (g._2284bp—2333bp_ ins GGTACGTAACCCAAATAATATTTCACTGTCTGTTCGCTGATCATCCAACTCAA) compared to that of the dark strain. The insertion results in alterations to the amino acid sequence during the translation process. Moreover, two mutation sites are identified within the intronic regions. In the 13th intron, the white strain exhibits a deletion of 5 bases located between 1,910 bp and 1,914 bp (g._1910bp-1914bp_ del ACTCG). In the 14th intron, a 3-base substitution is observed in the white strain, occurring between 2,039 bp and 2,041 bp (g._2039bp—2041bp_ AAC > TCG). Two mutations are identified within the promoter region. In the white strain, a four-base insertion mutation occurs between −1,298 bp and −1,301 bp (g._-1298bp—-1301bp_ ins AGTA). Additionally, a nine-base insertion mutation is observed between −1,317 bp and −1,325 bp in the white strain (g._-1317bp—-1325bp_ ins CTTGACCTG) (Fig. 6). These sequence variations may contribute to the differences in cap color between the two parental strains, further supporting the critical role of the *pcmfs* gene in controlling cap color formation in *P. cornucopiae*.

## DISCUSSION

Oyster mushrooms are one of the most widely cultivated edible fungi in the world, valued for their nutritional benefits and delicious taste (24, 35). Since darker-colored oyster mushrooms are more favored by consumers, developing varieties with darker caps holds significant market potential. Identifying key genes that control cap color can accelerate the breeding process for darker-colored oyster mushroom varieties.

The pigment in *Pleurotus* spp. is melanin, which is a significant fungal pigment (12, 36). However, due to the complex genetic background of edible mushrooms, only a few functional genes related to melanin synthesis have been reported. Gao et al. identified two genes, *ppo6* and *4cl_9*, which are associated with melanin synthesis and may be involved in cap color formation in *A. bisporus* (26, 37). Wang et al. identified the gene *hm01g09135*, encoding cytochrome *p450*, as an important candidate gene potentially involved in the formation of cap color in *Hypsizygus marmoreus* (38). Additionally, Ma et al. conducted multi-omics analysis and fine mapping on the fruiting body color of *Auricularia cornea* and identified *acvea* as the major gene controlling the color of the fruiting body (39). In our previous research, a 0.8 Mb major QTL region controlling cap color of *P. cornucopiae* was identified (34). Additionally, an important candidate gene, *pctyr*, which plays a crucial role in melanin formation, was further identified through comparative transcriptomic analysis. In this study, the gene controlling cap color of oyster mushroom was further mined based on the results of QTL mapping and comparative transcriptome analysis of previous research. As a result, *pcmfs* was identified as another key candidate gene. Expression pattern analysis and genetic transformation testing demonstrated that *pcmfs* palyed a critical role involved in the cap color formation in *P. cornucopiae*. Sequence analysis revealed that *pcmfs* encodes a protein belonging to the major facilitator superfamily (MFS). The MFS is one of the largest known superfamilies of membrane transport proteins, capable of facilitating the transmembrane transport of solutes such as carbohydrates, organic anions, inorganic anions, and peptides across electrochemical gradients (39–41). Members of the MFS typically possess 12 transmembrane helix units (42, 43). Through a unique folding mechanism, these proteins can adopt conformations with openings either facing the cytoplasm or the extracellular space, thereby operating via a “rocking switch” mechanism to mediate solute transport (44–46). In this study, protein structural analysis indicated that the PCMFS also features the typical 12 transmembrane helices. Subcellular localization results demonstrated that this gene is localized to the cell membrane, suggesting that it plays a crucial role in transmembrane transport.

In order to further explore the function of *pcmfs,* the expression of RNA products of *pcmfs* was analyzed by qPCR. The results revealed that the expression level of *pcmfs* in dark strains is over 1,000 times higher than that of white strains. Additionally, the expression patterns of this gene across different developmental stages and anatomical parts of the fruiting bodies showed a significant correlation with cap color. Specifically, the gene expression was significantly increased in the developmental stages with a darker color (young fruiting body stage) and parts with a darker color (pileipellis of fruiting body). This significant correlation indicated that the *pcmfs* gene may play an essential role in the formation of cap color in *P. cornucopiae*. Moreover, the genetic transformation experiments revealed that the cap color of the *pcmfs* overexpression strains is significantly darker compared to the wild-type strains, providing further evidence that *pcmfs* is a critical gene in cap color formation in *P. cornucopiae*. However, the cap color of the RNAi-PcMFS transformants was not significantly lighter than that of the WT strains as expected. The result of fruiting test showed that the cap color of the RNAi-PcMFS transformants is lighter than that of the WT strain albeit not to a significant degree (Fig. S1). The potential cause may lie in the employment of RNA interference, which, while partially reducing the expression level of the *pcmfs* gene, failed to achieve complete gene knockout, resulting in an insignificant impact on the phenotype. Additionally, previous studies have shown that cap color was controlled by multiple genes (47), and there might be compensatory effects from other genes. In the future, we will try to use gene editing technology to knock out the *pcmfs* gene and further validate its gene function. Overall, the results of our study are consistent with previous research. Reports have demonstrated the important role of MFS genes in melanin synthesis. For instance, Yuan et al. (48) identified the MFS gene as a key component in the melanin synthesis gene cluster of plant endophytic mycorrhizal fungi, involved in transporting melanin from the cytoplasm to the cell wall (48). Similarly, Ward et al. (49) found that MFS genes are crucial for melanin formation in fruit fly pupae, with gene knockout leading to a loss of the black phenotype and resulting in a white coloration (49). Based on these findings, it is hypothesized that *pcmfs* facilitates the extracellular transport of melanin granules to the cell wall, thereby playing a pivotal role in melanin synthesis in *P. cornucopiae*. To our knowledge, this is the first reported gene that may be involved in the melanin transport in edible fungi.

Comparative analysis of the *pcmfs* gene and its promoter sequences between the dark and white parent strains revealed a 53 bp insertion in the last exon of the *pcmfs* gene in the white parent strain. This insertion induces a frameshift mutation, which is likely a major factor contributing to the observed color difference. Moreover, two mutation sites were identified within the intronic regions. In the 13th intron, the white strain exhibited a deletion of 5 bases, while a 3-base substitution was observed in the 14th intron of the white strain. Previous research has demonstrated that sequence variations can influence gene expression. For instance, Waltz et al. (2013) found that deletions of several base pairs in the tyrosinase gene, a rate-limiting enzyme for melanin synthesis in *A. bisporus,* led to a 30% reduction in enzyme activity and consequently decreased melanin production (50). Additionally, the promoter sequence of the white parent strain exhibits insertions of 4 bp and 9 bp. Although the promoter does not directly code for proteins, it can affect gene expression (34). Thus, it is hypothesized that these sequence variations may influence the expression of the *pcmfs* gene.

In conclusion, this study identified a crucial gene, *pcmfs*, involved in the cap color formation in oyster mushroom. Unlike previously reported genes, *pcmfs* appears to play a crucial role in the extracellular transport of melanin granules to the cell wall. According to the previous reports, the pigment in *Pleurotus* species is melanin (12). In addition, the genetic background of *Pleurotus* species is similar (47). Therefore, we speculate that the *pcmfs* gene may also play an important role in the cap color formation in other *Pleurotus* species, but further experimental verification is needed. Both tyrosinase (*pctyr*) and *pcmfs* are important genes for cap color formation in *P. cornucopiae*. Co-expression of the two genes may change the mycelia color and cap color of white strains, but further verification is needed due to the complexity of melanin synthesis pathway.

This study is the first to report a gene responsible for melanin transport in edible fungi. This study will contribute to revealing the genetic mechanism of cap color formation in mushrooms, thereby facilitating molecular breeding for cap color traits.

## MATERIALS AND METHODS

### Strains and culture conditions

*P. cornucopiae* CCMSSC00406 with a grayish black cap color and *P. cornucopiae* CCMSSC00358 with a white cap color were selected as heterokaryotic parental strains. A hybrid strain HBPC067 with black caps was generated by crossing BPC067 (a single spore isolation of strain CCMSSC00406) and CCMSSC00358P1 (a constituent proto-clone of strain 358). F2 population was generated by crossing 175 single spore isolates that randomly selected from mature fruiting bodies of strain HBPC067 with tester line CCMSSC00358P2. Total four dark-colored strains (F2-48, F2-126, F2-131, F2-133) and four white strains (F2-14, F2-51, F2-70, F2-71) were selected from F2 population to use in this study. These strains were generated in a previous study (34) and deposited in China Center for Mushroom Spawn Standards and Control.

The WT and mutant strains were cultured on potato dextrose agar (PDA) plates, *Escherichia coli* (DH5α) was cultured in Luria–Bertani (LB) medium containing 50 ng/mL kanamycin, and *Agrobacterium tumefaciens* (GV3101) was cultured in LB containing 50 ng/mL kanamycin and 25 ng/mL rifampicin. The PDA culture medium was purchased from Beijing Boidee Biotechnology Co., Ltd. (Beijing, China), Sensory cells purchased from TransGen Biotech (Beijing, China). Restrictive endonuclease was purchased from New England Biolabs (NEB) (Beverly, MA, USA), and DNA polymerase, reverse transcription kits, and DNA gel extraction kits were purchased from Vazyme (Nanjing, China). Primer synthesis and DNA sequencing were completed by Tsingke Biotech (Xian, China). The plasmid pBI121-EGFP was purchased from Miaoling. Biology (Wuhan, China).

Mushroom cultivation was conducted as previously reported. Mushrooms were grown in 280 mL polypropylene bottles, each containing 140 g of a pre-mixed substrate comprising 94% cotton seed hull, 5% wheat bran, and 1% gypsum, adjusted to a moisture content of 65% with tap water. Four replicates were inoculated for each strain. First, the strains were cultured on potato dextrose agar (PDA) medium at 28°C for 7 days. Afterward, three pieces of mycelium (1 cm^2^) was inoculated into each bottle. Mycelia achieved full colonization in the bottle within 4 weeks of incubation at 25°C in the dark. And then, the bottles were moved to a fruiting chamber with controlled conditions set at 16–18°C, 80%–90% relative humidity, and a 12 h photoperiod with light intensity maintained between 300 and 350 lux. The CO_2_ concentration was consistently kept below 1,000 ppm through effective ventilation. After that, the primordium appears about 7 days later. Finally, it takes about 5 days from primordium to mature fruiting body.

### Identification, cloning, and sequence analysis of *pcmfs* genes

Primers of *pc*mfs-F/*pc*mfs-R and Pro-F/Pro-R were designed to amplify genic and promoter regions, respectively, using the Ex Taq enzyme (Takara, Dalian, China). PCR products were cloned using the pEASY-T1 Cloning Kit (TRANSGEN BIOTECH, Beijing) and then subjected to Sanger sequencing to identify DNA polymorphisms. *pcmfs* sequence is available through online websites (http://www.bioinformatics.org/sms/index.html). The Conserved Domain Search Service (CD Search) (https://www.ncbi.nlm.nih.gov/Structure/cdd/wrpsb.cgi) was used to predict the conserved domain of the PCMFS protein. DNAMAN 6.0 software was used for multiple sequence alignment. The protein structural and topology of PcMFS was predicted through PHYRE2 (http://www.sbg.bio.ic.ac.uk/phyre2/html/page.cgi?id=index) and HMMTOP (https://cctop.ttk.hu/). All primers used in this study are shown in Table S1.

### Subcellular localization of *pcmfs*

The *pcmfs* genes were cloned and inserted into the vector pBI121-EGFP and CBL1-RFP (a membrane localization protein), respectively, through homologous recombination and then introduced into *A. tumefaciens* EHA105, with empty space as the control. Afterward, *A. tumefaciens* EHA105 containing the target plasmid was cultured, collected, and suspended in a concentrated solution (150 mM acetylsyringone, 10 mM MES monohydrate, 10 mM magnesium chloride, and pH 5.6) to achieve a final OD value of 1.0. Finally, *A. tumefaciens* was injected into tobacco leaves with good growth and cultured for 3 days. The results were observed and recorded using a confocal microscope.

### Quantitative PCR

Total RNA was extracted using the E.Z.N.A. Plant RNA Kit (Omega Bio-Tek, Norcross, GA, USA) and subsequently converted to cDNA with a reverse transcription kit HiScript Q RT SuperMix for qPCR (Vazyme, Biotech). qPCRs were performed with AceQ qPCR SYBR Green Master Mix (Vazyme, Biotech) and the ABI 7500 Real-Time PCR amplifier (Applied Biosystems, Foster City, CA, USA). *β-tubulin* gene was used as the internal reference, and qPCR was used to analyze the specific mRNA expression level of the *pcmfs* gene. The qPCR amplification program was as follows: amplification at 95°C for 3 min, amplification at 95°C for 3 s, amplification at 60°C for 32 s, 40 cycles, and amplification at 72°C for 3 s. The relative gene expression was analyzed according to the 2*^−^*^∆∆CT^ method. Sequences of the primers used for qPCR are listed in Table S1.

### Construction of overexpression and RNA interference plasmids and strains

A modified pCAMBIA1300 vector containing the hygromycin B phosphotransferase gene (*hyg*) was used to construct OE and RNAi plasmids. The cDNA sequence of candidate gene pcmfs was amplified by PCR using the cDNA of strain CCMSSC00406 as a template. This fragment was cloned into the pCAMBIA1300 vectors to generate the overexpression plasmids. An RNAi-F sequence and a RNAi-R sequence were obtained by PCR using the cDNA of CCMSSSC00406, and the two sequences were connected in the interference vector. Finally, the OE and RNAi were transferred to the WT strains by using *Agrobacterium tumefaciens* GV3101 according to a previous study, i.e., the dark strain CCMSSC00406 was selected on complete yeast medium (CYM) containing 90 mg/mL hygromycin B. The transformants were screened preliminarily by PCR analysis of the *hyg* fragment. Next, three independent transformants with the highest overexpression efficiency or silencing efficiency were selected based on qPCR analysis. The primers used to amplify target gene fragments are shown in Table S1.

The OE and RNAi transformants were cultivated to produce fruiting bodies as described in “2.1 Strains and Culture Conditions.” Three independent biological replicates were cultivated for each transformant. The cap color of each transformant was observed.

### Extraction and detection of melanin

The melanin of WT strain and OE-PcMFS transformants was extracted and detected according to an approach reported previously (12). Initially, the caps of the fruiting bodies were ground using a homogenizer and combined with a 1:30 vol ratio of mushroom mash to 1.5 M NaOH solution at 25°C. The prepared mixture underwent sonication (Fisher Scientific, USA) at 80 W for 80 min to ensure thorough extraction. Subsequently, the mixture was clarified by centrifugation at 10,000 rpm for 15 min. The supernatant was transferred into an Erlenmeyer flask, where its pH was adjusted to 1.5 with a 7 M HCl solution. After pH adjustment, the supernatant was incubated at room temperature for 3 h to promote the formation of precipitates. The precipitate was collected by centrifugation at 10,000 rpm for 15 min and thoroughly washed with deionized water until a neutral pH was reached. Finally, the crude pigments were obtained through dehydration using a freeze dryer (Delta 1–24 LSC, Germany).

### Data analysis

All tests included at least three biological replicates, and the data are presented as the mean ± SEM. GraphPad Prism 6 (GraphPad Software, Inc., San Diego, CA, USA) and SPSS 20.0 software (SPSS Inc., Chicago, IL, USA) were used for the statistical analyses. One-way analysis of variance (ANOVA) was used for multiple comparisons. Statistical significance is represented by different letters corresponding to *P* < 0.05 based on the Duncan’s test.

## Data Availability

The sequence of *pcmfs* has been deposited in GenBank under accession number PQ757328.

## SUPPLEMENTAL MATERIAL

Figure S1 aem.01894-24-s0001.docx Construction of RNAi-PcMFS transformants and cap color phenotype images.

Table S1 aem.01894-24-s0002.xlsx Primers used in this study.

ASM does not own the copyrights to Supplemental Material that may be linked to, or accessed through, an article. The authors have granted ASM a non-exclusive, world-wide license to publish the Supplemental Material files. Please contact the corresponding author directly for reuse.

## References

[1] Yang Z, Xu J, Fu Q, Fu X, Shu T, Bi Y, Song B. 2013. Antitumor activity of a polysaccharide from Pleurotus eryngii on mice bearing renal cancer. Carbohydr Polym 95:615–620. doi:10.1016/j.carbpol.2013.03.024 23648020

[2] Bellettini MB, Fiorda FA, Maieves HA, Teixeira GL, Ávila S, Hornung PS, Júnior AM, Ribani RH. 2019. Factors affecting mushroom Pleurotus spp. Saudi J Biol Sci 26:633–646. doi:10.1016/j.sjbs.2016.12.005 31048986 PMC6486501

[3] Knop D, Yarden O, Hadar Y. 2015. The ligninolytic peroxidases in the genus Pleurotus: divergence in activities, expression, and potential applications. Appl Microbiol Biotechnol 99:1025–1038. doi:10.1007/s00253-014-6256-8 25503316

[4] Qu J, Huang C, Zhang J. 2016. Genome-wide functional analysis of SSR for an edible mushroom Pleurotus ostreatus. Gene 575:524–530. doi:10.1016/j.gene.2015.09.027 26386282

[5] Chun-Ge S, Chen-Yang H, Qiang C, Tolgor B, Jin-Xia Z. 2012. Fruit-body color inheritance of Pleurotus cornucopiae. Sci Agric Sinica 45:3124–3129. doi:10.3864/j.issn.0578-1752.2012.15.014

[6] Royse D. 2004. Yield, mushroom size and time to production of Pleurotus cornucopiae (oyster mushroom) grown on switch grass substrate spawned and supplemented at various rates. Bioresour Technol 91:85–91. doi:10.1016/S0960-8524(03)00151-2 14585625

[7] Wang X-M, Zhang J, Wu L-H, Zhao Y-L, Li T, Li J-Q, Wang Y-Z, Liu H-G. 2014. A mini-review of chemical composition and nutritional value of edible wild-grown mushroom from China. Food Chem 151:279–285. doi:10.1016/j.foodchem.2013.11.062 24423533

[8] Velíšek J, Cejpek K. 2011. Pigments of higher fungi - a review. Czech J Food Sci 29:87–102. doi:10.17221/524/2010-CJFS

[9] Wu Z, Zhang M, Yang H, Zhou H, Yang H. 2018. Production, physico-chemical characterization and antioxidant activity of natural melanin from submerged cultures of the mushroom Auricularia auricula. Food Biosci 26:49–56. doi:10.1016/j.fbio.2018.09.008

[10] Ribera J, Panzarasa G, Stobbe A, Osypova A, Rupper P, Klose D, Schwarze F. 2019. Scalable biosynthesis of melanin by the basidiomycete Armillaria cepistipes. J Agric Food Chem 67:132–139. doi:10.1021/acs.jafc.8b05071 30541276

[11] Weijn A, Bastiaan-Net S, Wichers HJ, Mes JJ. 2013. Melanin biosynthesis pathway in Agaricus bisporus mushrooms. Fungal Genet Biol 55:42–53. doi:10.1016/j.fgb.2012.10.004 23123422

[12] Zhang Y, Wu X -r., Huang C, Zhang Z, Gao W. 2022. Isolation and identification of pigments from oyster mushrooms with black, yellow and pink caps. Food Chem 372:131171. doi:10.1016/j.foodchem.2021.131171 34601416

[13] Liu R, Meng X, Mo C, Wei X, Ma A. 2022. Melanin of fungi: from classification to application. World J Microbiol Biotechnol 38:228. doi:10.1007/s11274-022-03415-0 36149606

[14] Valiante V, Jain R, Heinekamp T, Brakhage AA. 2009. The MpkA MAP kinase module regulates cell wall integrity signaling and pyomelanin formation in Aspergillus fumigatus. Fungal Genet Biol 46:909–918. doi:10.1016/j.fgb.2009.08.005 19715768

[15] Cao W, Zhou X, McCallum NC, Hu Z, Ni QZ, Kapoor U, Heil CM, Cay KS, Zand T, Mantanona AJ, Jayaraman A, Dhinojwala A, Deheyn DD, Shawkey MD, Burkart MD, Rinehart JD, Gianneschi NC. 2021. Unraveling the structure and function of melanin through synthesis. J Am Chem Soc 143:2622–2637. doi:10.1021/jacs.0c12322 33560127

[16] Maranduca MA, Branisteanu D, Serban DN, Branisteanu DC, Stoleriu G, Manolache N, Serban IL. 2019. Synthesis and physiological implications of melanic pigments. Oncol Lett 17:4183–4187. doi:10.3892/ol.2019.10071 30944614 PMC6444329

[17] Nosanchuk JD, Stark RE, Casadevall A. 2015. Fungal melanin: what do we know about structure? Front Microbiol 6:1463. doi:10.3389/fmicb.2015.01463 26733993 PMC4687393

[18] Apaza Ticona L, Thiebaut Estrada C, Rumbero Sánchez Á. 2021. Inhibition of melanin production and tyrosinase activity by flavonoids isolated from Loranthus acutifolius. Nat Prod Res 35:4690–4693. doi:10.1080/14786419.2019.1709185 31894701

[19] Niki Y, Adachi N, Fukata M, Fukata Y, Oku S, Makino-Okamura C, Takeuchi S, Wakamatsu K, Ito S, Declercq L, Yarosh DB, Mammone T, Nishigori C, Saito N, Ueyama T. 2023. S-palmitoylation of tyrosinase at cysteine^500^ regulates melanogenesis. J Invest Dermatol 143:317–327. doi:10.1016/j.jid.2022.08.040 36063887

[20] Ullah S, Park C, Ikram M, Kang D, Lee S, Yang J, Park Y, Yoon S, Chun P, Moon HR. 2019. Tyrosinase inhibition and anti-melanin generation effect of cinnamamide analogues. Bioorg Chem 87:43–55. doi:10.1016/j.bioorg.2019.03.001 30856375

[21] Fan X, Zhang P, Batool W, Liu C, Hu Y, Wei Y, He Z, Zhang SH. 2023. Contribution of the tyrosinase (MoTyr) to melanin synthesis, conidiogenesis, appressorium development, and pathogenicity in Magnaporthe oryzae. J Fungi (Basel) 9:311. doi:10.3390/jof9030311 36983479 PMC10059870

[22] Bayry J, Beaussart A, Dufrêne YF, Sharma M, Bansal K, Kniemeyer O, Aimanianda V, Brakhage AA, Kaveri SV, Kwon-Chung KJ, Latgé JP, Beauvais A. 2014. Surface structure characterization of Aspergillus fumigatus conidia mutated in the melanin synthesis pathway and their human cellular immune response. Infect Immun 82:3141–3153. doi:10.1128/IAI.01726-14 24818666 PMC4136205

[23] Upadhyay S, Xu X, Lowry D, Jackson JC, Roberson RW, Lin X. 2016. Subcellular compartmentalization and trafficking of the biosynthetic machinery for fungal melanin. Cell Rep 14:2511–2518. doi:10.1016/j.celrep.2016.02.059 26972005 PMC4805463

[24] Toledo AV, Franco MEE, Yanil Lopez SM, Troncozo MI, Saparrat MCN, Balatti PA. 2017. Melanins in fungi: types, localization and putative biological roles. Physiol Mol Plant Pathol 99:2–6. doi:10.1016/j.pmpp.2017.04.004

[25] Foulongne-Oriol M, Rodier A, Rousseau T, Savoie JM. 2012. Quantitative trait locus mapping of yield-related components and oligogenic control of the cap color of the button mushroom, Agaricus bisporus. Appl Environ Microbiol 78:2422–2434. doi:10.1128/AEM.07516-11 22267676 PMC3302609

[26] Gao W, Weijn A, Baars JJP, Mes JJ, Visser RGF, Sonnenberg ASM. 2015. Quantitative trait locus mapping for bruising sensitivity and cap color of Agaricus bisporus (button mushrooms). Fungal Genet Biol 77:69–81. doi:10.1016/j.fgb.2015.04.003 25881912

[27] Im CH, Park Y-H, Hammel KE, Park B, Kwon SW, Ryu H, Ryu J-S. 2016. Construction of a genetic linkage map and analysis of quantitative trait loci associated with the agronomically important traits of Pleurotus eryngii. Fungal Genet Biol 92:50–64. doi:10.1016/j.fgb.2016.05.002 27166667

[28] Larraya LM, Alfonso M, Pisabarro AG, Ramírez L. 2003. Mapping of genomic regions (quantitative trait loci) controlling production and quality in industrial cultures of the edible basidiomycete Pleurotus ostreatus. Appl Environ Microbiol 69:3617–3625. doi:10.1128/AEM.69.6.3617-3625.2003 12788770 PMC161546

[29] Zhang Y, Gao W, Sonnenberg A, Chen Q, Zhang J, Huang C. 2021. Genetic linkage and physical mapping for an oyster mushroom. Appl Environ Microbiol 87:e00953–21. doi:10.1128/AEM.00953-21 34406836 PMC8516045

[30] Huang Z, Peng G, Liu X, Deora A, Falk KC, Gossen BD, McDonald MR, Yu F. 2017. Fine mapping of a clubroot resistance gene in chinese cabbage using SNP markers identified from bulked segregant RNA sequencing. Front Plant Sci 8:1448. doi:10.3389/fpls.2017.01448 28894454 PMC5581393

[31] Yang J, Jiang H, Yeh CT, Yu J, Jeddeloh JA, Nettleton D, Schnable PS. 2015. Extreme-phenotype genome-wide association study (XP-GWAS): a method for identifying trait-associated variants by sequencing pools of individuals selected from A diversity panel. Plant J 84:587–596. doi:10.1111/tpj.13029 26386250

[32] Zhu Y, Li Q. 2024. Mitf involved in shell pigmentation by activating tyrosinase-mediated melanin synthesis in pacific oyster (Crassostrea gigas). Gene 897:148086. doi:10.1016/j.gene.2023.148086 38104952

[33] Zhu Y, Li Q, Yu H, Liu S, Kong L. 2022. Expression of tyrosinase-like protein genes and their functional analysis in melanin synthesis of Pacific oyster (Crassostrea gigas). Gene 840:146742. doi:10.1016/j.gene.2022.146742 35868415

[34] Zhang Y, Huang C, van Peer AF, Sonnenberg ASM, Zhao M, Gao W. 2022. Fine mapping and functional analysis of the gene pctyr, involved in control of cap color of Pleurotus cornucopiae. Appl Environ Microbiol 88:e0217321. doi:10.1128/aem.02173-21 35289641 PMC9004381

[35] Golovko O, Kaczmarek M, Asp H, Bergstrand KJ, Ahrens L, Hultberg M. 2022. Uptake of perfluoroalkyl substances, pharmaceuticals, and parabens by oyster mushrooms (Pleurotus ostreatus) and exposure risk in human consumption. Chemosphere 291:132898. doi:10.1016/j.chemosphere.2021.132898 34780735

[36] Selvakumar P, Rajasekar S, Periasamy K, Raaman N. 2023. Isolation and characterization of melanin pigment from Pleurotus cystidiosus (telomorph of Antromycopsis macrocarpa). World J Microbiol Biotechnol 24:2125–2131. doi:10.1007/s11274-008-9718-2

[37] Eisenman HC, Casadevall A. 2012. Synthesis and assembly of fungal melanin. Appl Microbiol Biotechnol 93:931–940. doi:10.1007/s00253-011-3777-2 22173481 PMC4318813

[38] Wang G, Chen L, Tang W, Wang Y, Zhang Q, Wang H, Zhou X, Wu H, Guo L, Dou M, Liu L, Wang B, Lin J, Xie B, Wang Z, Liu Z, Ming R, Zhang J. 2021. Identifying a melanogenesis-related candidate gene by a high-quality genome assembly and population diversity analysis in Hypsizygus marmoreus. J Genet Genomics 48:75–87. doi:10.1016/j.jgg.2021.01.002 33744162

[39] Ma X, Lu L, Zhang Y, Fang M, Shao K, Sun X, Yao F, Wang P. 2023. Velvet family members regulate pigment synthesis of the fruiting bodies of Auricularia cornea. J Fungi (Basel) 9:412. doi:10.3390/jof9040412 37108867 PMC10140996

[40] Yan N. 2015. Structural biology of the major facilitator superfamily transporters. Annu Rev Biophys 44:257–283. doi:10.1146/annurev-biophys-060414-033901 26098515

[41] Sauve S, Williamson J, Polasa A, Moradi M. 2023. Ins and outs of rocker switch mechanism in major facilitator superfamily of transporters. Membranes (Basel) 13:462. doi:10.3390/membranes13050462 37233523 PMC10221096

[42] Zhang Y, Hu T, Zhou H, Zhang Y, Jin G, Yang Y. 2016. Antidiabetic effect of polysaccharides from Pleurotus ostreatus in streptozotocin-induced diabetic rats. Int J Biol Macromol 83:126–132. doi:10.1016/j.ijbiomac.2015.11.045 26627601

[43] Zhang XC, Zhao Y, Heng J, Jiang D. 2015. Energy coupling mechanisms of MFS transporters. Protein Sci 24:1560–1579. doi:10.1002/pro.2759 26234418 PMC4594656

[44] Drew D, North RA, Nagarathinam K, Tanabe M. 2021. Structures and general transport mechanisms by the major facilitator superfamily (MFS). Chem Rev 121:5289–5335. doi:10.1021/acs.chemrev.0c00983 33886296 PMC8154325

[45] Wu H-H, Symersky J, Lu M. 2020. Structure and mechanism of a redesigned multidrug transporter from the major facilitator superfamily. Sci Rep 10:3949. doi:10.1038/s41598-020-60332-8 32127561 PMC7054563

[46] Yan N. 2013. Structural advances for the major facilitator superfamily (MFS) transporters. Trends Biochem Sci 38:151–159. doi:10.1016/j.tibs.2013.01.003 23403214

[47] Zhang Y, Gao W, Sonnenberg A, Chen Q, Zhang J, Huang C. 2021. Genetic linkage and physical mapping for an oyster mushroom (Pleurotus cornucopiae) and quantitative trait locus analysis for cap color. Appl Environ Microbiol 87:e0095321. doi:10.1128/AEM.00953-21 34406836 PMC8516045

[48] Yuan Z, Druzhinina IS, Gibbons JG, Zhong Z, Van de Peer Y, Rodriguez RJ, Liu Z, Wang X, Wei H, Wu Q, Wang J, Shi G, Cai F, Peng L, Martin FM. 2021. Divergence of a genomic island leads to the evolution of melanization in a halophyte root fungus. ISME J 15:3468–3479. doi:10.1038/s41396-021-01023-8 34108667 PMC8629976

[49] Ward CM, Aumann RA, Whitehead MA, Nikolouli K, Leveque G, Gouvi G, Fung E, Reiling SJ, Djambazian H, Hughes MA, Whiteford S, Caceres-Barrios C, Nguyen TNM, Choo A, Crisp P, Sim SB, Geib SM, Marec F, Häcker I, Ragoussis J, Darby AC, Bourtzis K, Baxter SW, Schetelig MF. 2021. White pupae phenotype of tephritids is caused by parallel mutations of a MFS transporter. Nat Commun 12:491. doi:10.1038/s41467-020-20680-5 33479218 PMC7820335

[50] Waltz E. 2016. Gene-edited CRISPR mushroom escapes US regulation. Nature 532:293. doi:10.1038/nature.2016.19754 27111611

