# Supplementary material for: Identification and functional characterization of major gene pcmfs, controlling cap color formation in Pleurotus cornucopiae
Source: Appl Environ Microbiol. 2025 Feb 27;91(3):e01894-24. doi: 10.1128/aem.01894-24 (PMC11921331; doi:10.1128/aem.01894-24)
Supplement: Figure S1 — Construction of RNAi-PcMFS transformants and cap color phenotype images. [file aem.01894-24-s0001.docx]

**Figure S1**


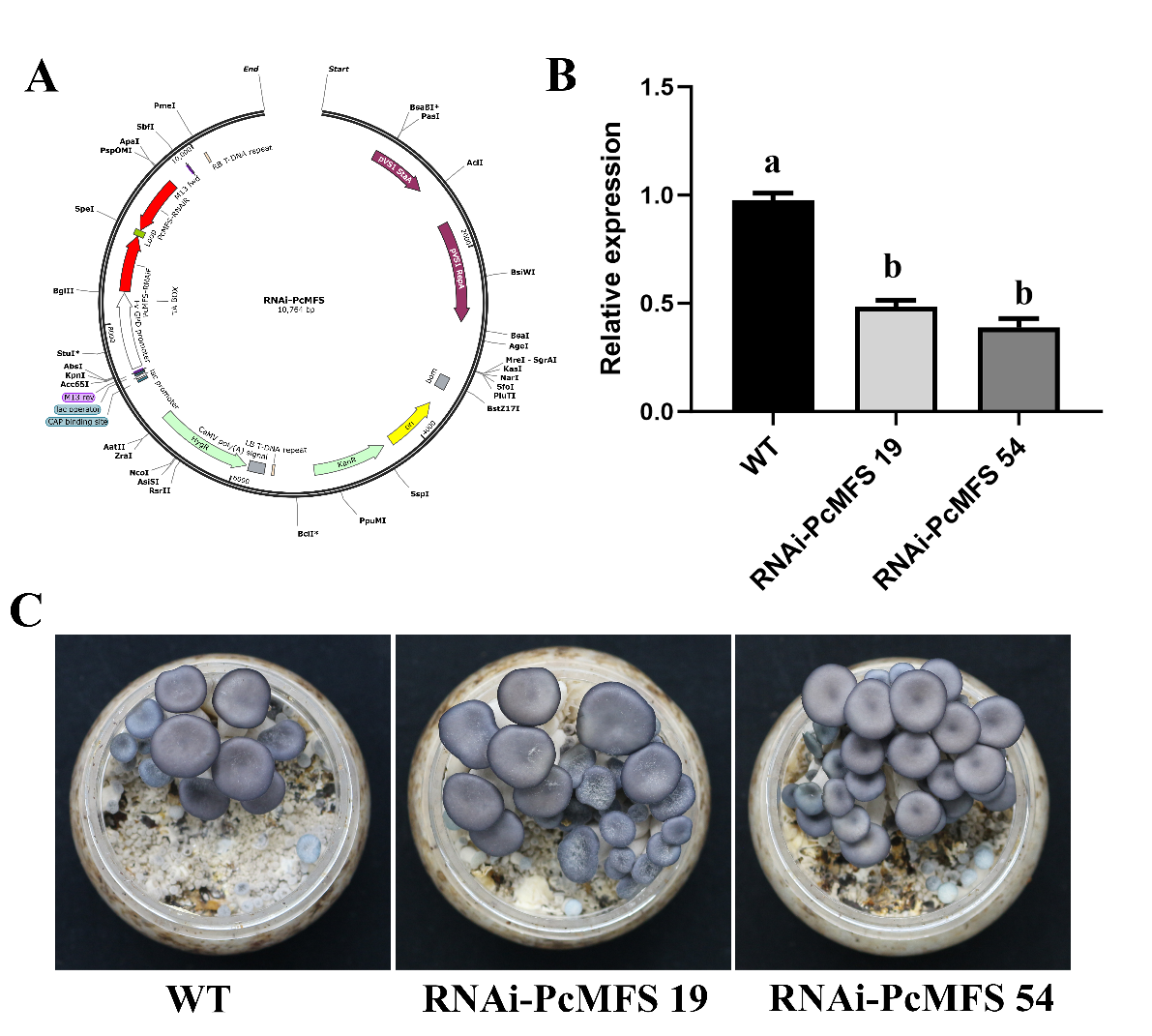


**Figure S1** Construction of RNAi-PcMFS transformants and cap color phenotype images. (A) Plasmid map of RNAi-PcMFS; (B) Expression levels of the *pcmfs* gene in RNAi-PcMFS transformants. qPCR results showed that the expression of pcmfs gene in strain RNAi-pcmfs 19 and RNAi-pcmfs 54 was down-regulated by 48.98% and 42.86%, respectively, compared with that in WT strains. The expression level of *pcmfs* in wild-type strain is defined as 1.; (C) Cap color phenotype images of wild-type strain and RNAi-PcMFS transformants. the cap color of the RNAi-PcMFS transformants is lighter than that of the WT strain, albeit not to a significant degree. Data are presented as means ± SD (n=3). Different letters indicate significant differences between treatments (P<0.05, Duncan’s multiple range test)
